# HPLC-ESI/MS-MS characterization of compounds in *Dolomiaea costus* extract and evaluation of cytotoxic and antiviral properties: molecular mechanisms underlying apoptosis-inducing effect on breast cancer

**DOI:** 10.1186/s12906-023-04164-9

**Published:** 2023-10-06

**Authors:** Heba A. S. El-Nashar, Omayma A. Eldahshan, Nasra F Abdel Fattah, Samah A Loutfy, Ibrahim M Abdel-Salam

**Affiliations:** 1 Department of Pharmacognosy, Faculty of Pharmacy, Ain Shams University, Abbassia, 11566, Cairo, Egypt; 2 Centre of Drug Discovery Research and Development, Ain Shams University, Cairo, Egypt; 3 Virology & Immunology Unit, Cancer Biology Dept, National Cancer Institute (NCI), Cairo University, Fom El-Khalig 11796, Cairo, Egypt; 4 Nanotechnology research center, the British University in Egypt, Suez Desert Road, P.O. Box 43, El-Shorouk City, Cairo, 11837 Egypt; 5 Biochemistry and Molecular Biology Unit, Cancer Biology Dept, National Cancer Institute (NCI), Cairo University, Fom El-Khalig 11796, Cairo, Egypt

**Keywords:** Antiviral, Apoptosis, Asteraceae, *Saussurea costus*, Flavonoids, Breast cancer

## Abstract

**Background:**

*Dolomiaea costus* (syn: *Saussurea costus*; Family Asteraceae) occupies an important place in the traditional Chinese medicinal plants and is prescribed for a wide range of disorders. The current study aimed to tentatively identify the phytoconstituents of *D. costus* extract and to explore antiproliferative activity against human breast cancer cells and its possible apoptotic mechanism along with antiviral activity against human adenovirus 5 (Adv-5).

**Methods:**

The phytoconstituents of 70% ethanol extract of *D. costus* were assessed using HPLC/ESI-MS/MS technique. The cell viability was investigated against breast cancer cell line (MCF-7) via 3-(4,5-dimethylthiazol-2-yl)-2,5-diphenyltetrazolium bromide (MTT) assay. Mechanistically, the apoptotic effects on the Bax, Bcl2 and Caspase 3 were determined via quantitative reverse transcriptase-polymerase chain reaction (RT-qPCR). Further, the antiviral activity was assessed against Adv-5 based on virucidal and adsorption mechanisms.

**Results:**

The HPLC/MS analysis of the extract revealed tentative identification of twenty compounds of polyphenolic nature, mainly flavonoids, lignans, coumarins, and anthocyanidins. The plant extract showed a cytotoxic effect against MCF-7 and Vero cells with IC_50_ values of 15.50 and 44 µg/ml, respectively, indicating its aggressiveness against the proliferation of breast cancer cells as confirmed by apoptotic genes expression which revealed upregulation of Bax and Caspase 3 but further insight analysis is needed to explore exact mechanistic pathway. Antiviral activity against Adv-5 was observed at a non-toxic concentration of the tested extract.

**Conclusions:**

Such observations against human breast cancer and viral replication supported further studies for nanoformulations in drug delivery systems as targeting therapy and in vivo studies before biomedical applications.

**Supplementary Information:**

The online version contains supplementary material available at 10.1186/s12906-023-04164-9.

## Introduction

The genus *Saussurea* (Fam. Asteraceae) comprises about 400 species distributed mainly in the north-temperate zone area including alpine habitats, the Himalayas, and Central Asia [1]. About 30 species have been recorded in Traditional Chinese Medicine (TCM), Tibetan medicines, Uyghur medicine, Mongolian medicine, and Kazakhstan medicine for broad-spectrum applications like anti-inflammatory, analgesic, antifatigue, anti-aging, hormonal-related gynecological disorders, infertility, and immunomodulation [2–4]. Among these species, *S. costus*, *S. involucrate*, *S. eopygmaea*, *S. obvallata*, *S. polycolea*, *S. laniceps*, and *S. medusa* have been used in both indigenous medical applications and current herbal therapies [5, 6].

*Dolomiaea costus* (syn. *Saussurea costus*) occupies an important place in the traditional Chinese medicinal plants [7–9]. It is well-known as a rich source of different bioactive phytoconstituents with reported interesting activities like flavonoids, phenylpropanoids, lignans, coumarins, monoterpenes, sesquiterpene lactones, steroids and volatile oils [10, 11]. In Arabian countries, *D. costus* is commonly named costus root, Kust and Qist Hindi. In the folk medicine, *D. costus* was prescribed for typhus fever, rheumatism, nervous disorders, irregular menstruation, heart diseases, asthma, gastric ulcer, inflammation, liver diseases and for hair to kill lice and to turn grey hair to black [12–14]. The essential oil isolated from *D. costus* possesses very strong aroma and is employed in high-grade perfumes, hair cosmetic formulation, and insect repellents [7]. Scientific evidence efforts proved that the *D. costus* roots displayed antioxidant, anxiolytic, hepatoprotective, antiulcer, hypolipidemic, anti-inflammatory, neuroprotective, antimicrobial, antiparasitic and antirheumatic properties [15–18]. Further, the methanol extract showed cardiotonic effects [19], while the petroleum ether extract showed potent anticonvulsant activity against picrotoxin-induced convulsions in mice [15, 20]. Interestingly, the phytochemicals isolated from *D. costus* like costunolide and dehydrocostuslactone were found to suppress tumor growth as well as metastases of breast cancer cell via tumor necrosis factor*-α*-(TNF-*α*)-induced nuclear factor-kappa (NF-κB) activation, leading to inhibition of MDA-MB-231 migration and invasion. Further, these compounds remarkedly reduced matrix metallopeptidase-9 (MMP-9) expression which is a well-known NF-kB-dependent gene and play a vital role to proceed breast cell cancer growth and metastases [21, 22]. Moreover, *D. costus* was found to be a potent inducer of apoptosis owing to the presence of costunolide as a major phytoconstituent [17, 23, 24].

Breast cancer remains the most common cause of cancer deaths among women globally, its treatment has many drawbacks and several adverse effects [25]. Recent studies have suggested many medicinal plants act as anti-cancer agents by inducing apoptosis in cancer cells [25–30]. *Costus* showed to have anti-hepatotoxic, anti-diabetic, antifungal, anthelmintic, anti-tumor, anti-inflammatory, anti-ulcer, antimicrobial, and immunostimulant effects. Previously, this species demonstrated its protective role against Ehrlich Solid Tumor (EST)-induced cardiac toxicity, injury, and alterations in apoptotic p53, pro-apoptotic Bax, and vascular endothelial growth factor (VEGF) expression [25]. Moreover, it demonstrated its ability to induce apoptosis in the breast and colon cancers [31]. This information encouraged us to do our research on this plant to investigate our own plant to obtain optimum concentration before proceeding nanotechnology-based drug delivery approach and in vivo study to manage such chronic disease.

Human adenovirus 5 (Adv-5) can affect multiple human organs such as gastrointestinal tract, respiratory tract, and ocular surface. The children and immunocompromised adults are more susceptible to adenoviral infections [32], also no FDA approved antivirals for such virus [33]. To our knowledge, there is no published data available for the utilization of *D. costus* root extract as antiviral agent against replication of human adenovirus type 5 in cell culture.

HPLC/ESI-MS/MS is well-known as a comprehensive analytical technique used for the identification of plant metabolites [34, 35]. In the literature of *D. costus*, we found extensive studies accomplished on its essential oil, while the other plant phytoconstituents have not been investigated in depth. Therefore, the current study was designed to tentatively identify the phytochemical constituents of 70% ethanol extract of *D. costus* using HPLC/ESI-MS/MS technique. In addition, we aimed to explore the in vitro antiproliferative activity against human breast cancer cells and its possible underlying mechanism along with its possible antiviral activity against replication of human adenovirus in Vero cells, this will open new era of biomedical application based on both anti-cancerous and antiviral activities.

## Materials and methods

### Plant material and extraction

*D. Costus* roots were purchased from local herbal market, Cairo, Egypt, in May 2021. The plant was authenticated by Professor Usama K. Abdel Hameed, Department of Botany, Faculty of Science, Ain Shams University, Cairo, Egypt. A voucher specimen (Number: PHG-P-SC-435) is deposited at the Pharmacognosy Department, Faculty of Pharmacy, Ain Shams University, Cairo, Egypt. The plant roots (1 kg) were chopped into small pieces and extracted with 70% ethanol (3 × 10 L). The pooled extracts were evaporated under reduced pressure at 55 °C until complete dryness to obtain 37 g of a brown material.

### HPLC-ESI/MS-MS conditions

The phytochemical analysis of *D. Costus* extract were assessed according to previously reported method using high-performance liquid chromatographic (HPLC) analysis joined with an ESI-MS/MS spectrometer detector [35]. This technique allowed tentative identification of phytoconstituents based on the molecular weights. The plant extract (100 µg/ml) was dissolved in methanol (HPLC-grade), then filtered via membrane disc (0.20 μm). Then, the filtrate (10 µL) was injected to HPLC/ESI-MS/MS. The used HPLC instrument has the following specifications: Waters® stocked with a reversed phase C-18 column (ACQUITY UPLC-BEH C-18, particle size ~ 1.7 μm, dimensions = 2.1 × 50 mm). Prior to injection, the mobile phase was filtered through membrane disc filter (0.2 μm) and sonicated. The elution run took 35 min using gradient elution (water and methanol acidified with 0.1% formic acid) with flow rate of 0.2 mL/min. On an XEVO TQD triple quadruple instrument, positive and negative ions were acquired using ESI-MS. Waters® Corporation, Milford, MA01757, U.S.A supplied the HPLC unit and mass spectrometer. The vacuum pump was provided by Edwards®, U.S.A. at desolvation temperatures of 150 and 440° C. The mass spectra were obtained using the software Maslynx 4.1 at ESI range *m/z* of 100–1000. In order to tentatively identify the obtained mass spectra, the peak retention time (t_R_) and their fragmentation pattern were compared with the reported data in the literature.

### Assessment of cell viability

The cytotoxic effect of plant extract was assessed against in vitro model of human breast cancer cells (MCF-7) and Vero cells as a model of normal cells using colorimetric 3-(4,5-dimethylthiazol-2-yl)-2,5-diphenyltetrazolium bromide (MTT) assay according to previously published protocol [36]. Vero and MCF-7 ATCC origin were purchased commercially from a holding company for biological products &vaccines (VACSERA) in Cairo, Egypt. Tissue culture reagents and required staffs are obtained from analysis company in Egypt (Gibco- Merelbeke, Belgium). MTT dye was purchased from Serva Electrophoresis GmbH, Heidelberg, Germany. The cell lines were maintained at virology & Immunology laboratory by subculturing at standard conditions, 37 °C with 5% CO_2_. The assay relies on reduction of tetrazolium salts into their insoluble formazan crystals that can be solubilized and measured spectrophotometrically. First, MCF-7cells were seeded in a 96-well plate with a density of 10^4^ cell/well and incubated overnight at 37 °C with 5% CO_2_. After that, the cells were treated with serial concentrations of the plant extract. After 48 h of incubation, 30 µl of MTT was added into each well and then incubated at 37 °C for 3 h. About 200 µl of dimethyl sulfoxide was added to each well to dissolve the insoluble formazan crystals. The absorbance was read at 570 nm using a multimode microplate reader. The CC_50_ was calculated using non-linear regression analysis, CC_50_ was determined with GraphPad Prism software version 6 [37].

$$Cell\,viability\,(\% ) = \frac{{{A_{test}}}}{{{A_{control}}}} \times 100$$

Whereas (A _test_) is the mean absorbance of the tested sample, and (A _control_) is the mean absorbance of the control sample. In each assay negative control (untreated cells) and positive control (DMSO) was included.

### Assessment of apoptotic effect against MCF-7 using quantitative RT-qPCR assay

The expression level of apoptotic genes on a mRNA transcriptional level was investigated according to previously published protocol [38, 39]. The data obtained from quantitative reverse transcriptase polymerase chain reaction (RT-qPCR) were analyzed using the comparative CT method [40]. RT-qPCR was performed using Power SYBR Green PCR Master Mix, which was obtained from Thermo Scientific, USA. The cells were seeded at a density of 5 × 10^4^ cells/ml into a six-well plate. After 24 h, the cells were treated with the polymeric materials and incubated for 24 h [41]. The cells were trypsinized and harvested, then cell pellets were collected and stored at -80 °C. Briefly, the difference in cycle threshold, ^Δ^CT, was determined as the difference between the tested gene and human GAPDH. Then, we obtained ^ΔΔ^CT by finding the difference between the two groups. The fold change (FC) was calculated as 2^−ΔΔCT^.

### Assessment of antiviral activity against human adenovirus type 5 (Adv-5)

The antiviral activity of the plant extract was assessed based on two different antiviral mechanisms including virucidal mechanism and adsorption mechanism. AdenoVirus was obtained from Virology Unit, School of Medicine in Kuwait. The virus was propagated into vero cells and titrated using PCR assay and used in the antiviral assay. Initially, cells were seeded in a 6-well plate with a density of 5 × 10^5^ cell/well and incubated overnight at 37 °C with 5% CO_2_. For investigation of virucidal mechanism, the Adv-5 was pre-incubated with 0.5 CC_50_ of plant extract for 1 h at 4 °C. After 24 h of seeding, the cells were infected with the pre-incubated virus. For investigation of adsorption mechanism, the cells were seeded for 24 h with 0.5 CC_50_ of plant extract. After that, the cells were infected with Adv-5 and incubated for 24 h at 37 °C and 5% CO_2_. Viral copies/ml was determined using real time PCR assay, and the results were compared with the viral control [33, 42]. Prior starting the antiviral activity assay, ADV-5 was propagated in Vero cells and investigated under an inverted microscope until 80–90% of CPE and cell lysis [43]. After that, the virus culture was subjected to viral nucleic acid extraction using QIAmp RNA extraction kit, (Qiagen,Valencia, USA). The extraction was done according to the manufacturer’s instructions. Then, 100 ng of DNA template was used in the PCR assays. Detection and quantification of Adv-5 viral load was done by real time detection system (Applied Biosystems 7500 Fast Real-time PCR) according to previously published protocol [33, 42, 43].

## Results and discussion

### Characterization of *D. costus* phytoconstituents using HPLC/ESI-MS/MS technique

**Fig. 1:**
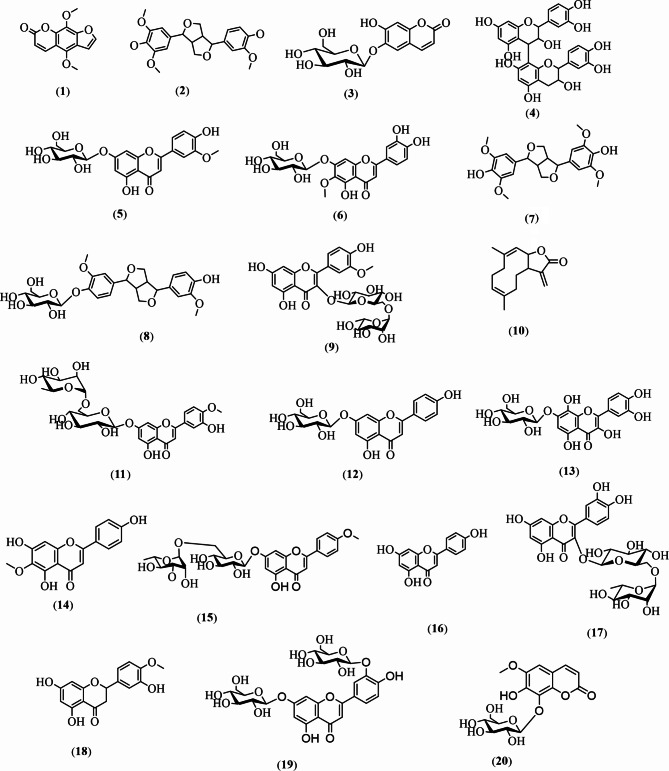
The chemical structures of identified compounds in *D. costus* extract using HPLC-MS/MS technique

The phytoconstituents of the 70% ethanol extract of *D. costus* were tentatively identified by HPLC/ESI-MS/MS and the total ion chromatogram (TIC) of metabolic profile is shown in supporting information (Fig. S1). The HPLC/ESI-MS/MS analysis of *D. costus* extract revealed the structural information for twenty peaks, identified according to the previous reported data. The retention times, pseudomolecular ions, molecular formula and fragments observed in MS/MS chromatograms are listed in Table 1. These compounds are distributed in four different main categories (flavonoids, lignans, coumarins, anthocyanidins, and sesquiterpene lactone). The identification of these compounds was based on matching the accurate mass (*m/z*) of the pseudomolecular [M-H]^−^ and [M+H]^−^ ions for the peaks and their fragmentation pattern with the previous reported data of literature. The chemical structures of the identified compounds are illustrated in Fig. 1.

**Table 1:** Characteristics of twenty peaks from *D. costus* tentatively identified by HPLC/ESI-MS/MS in the negative and positive ionization mode

Peak No	R_t_ (min)	(M – H) ^–^ *m/z*	(M + H) ^−^ *m/z*	Molecular weight	Molecular formula	MS^2^ fragments (*m/z*)	Tentative identification	Chemotaxonomy (Previous reported Species)	Class	Ref.
1.	0.22	245	247	246	C_13_H_10_O_5_	232, 217	Isopimpinellin	*Saussurea involucrata*	Coumarin	[3]
2.	0.74	387	389	388	C_21_H_24_O_7_	-	Medioresinol	*Saussurea stella*	Lignans	[1]
3.	1.04	339	-	340	C_15_H_16_O_9_	178	Esculetin hexoside	*Saussurea involucrata*	Coumarin	
4.	5.69	577	-	578	C_30_H_26_O_12_	407, 289, 125	Procyanidin B2	*Saussurea costus*	Anthocyanidin	[7]
5.	6.67	475	477	476	C_22_H_20_O_12_	300	Chrysoeriol-*O*-hexouronide	*Saussurea costus*	Flavonoid	[7]
6.	6.80	477	-	478	C_22_H_22_O_12_	316	Nepetin-*O*-hexoside	*Saussurea involucrata*	Flavonoid	[44]
7.	6.91	-	419	418	C_22_H_26_O_8_	387, 371, 327, 181	Syringaresinol	*Saussurea stella*	Lignan	[1]
8.	7.00	519	-	520	C_26_H_32_O_11_	357, 327, 151	Pinoresinol-O-hexoside	*Saussurea stella*	Lignan	[1]
9.	7.04	623	-	624	C_28_H_32_O_16_	315, 299	Isorhamnetin-*O*-hexoside-*O*-deoxyhexoside	*Saussurea costus* *Saussurea involucrata*	Flavonoid	[7, 44]
10.	7.14	-	233	232	C_15_H_20_O_2_		Costunolide		Sesquiterpene lactone	[45]
11.	7.92	461	-	462	C_22_H_22_O_11_	300, 283, 232, 148	Diosmetin-*O*-hexoside	*Saussurea costus* *Saussurea stella*	Flavonoid	[1, 7]
12.	9.15	431	433	432	C_21_H_20_O_10_	269, 225, 151	Apigenin-*O*-hexoside	*Saussurea involucrata*	Flavonoid	[45]
13.	9.47	-	481	480	C_21_H_20_O_13_	319	Gossypetin-*O*-hexoside	*Saussurea costus*	Flavonoid	[7]
14.	11.65	299	-	300	C_16_H_12_O_6_	284	Hispidulin		Flavonoid	[46]
15.	13.30	591	-	592	C_28_H_32_O_14_	284, 242	Acacetin-*O*-hexoside-*O*-deoxyhexoside	*Saussurea costus*	Flavonoid	[7]
16.	15.12	269	-	270	C_15_H_10_O_5_	225, 165, 151	Apigenin	*Saussurea costus*	Flavonoid	[7]
17.	18.25	609	611	610	C_27_H_30_O_16_	465, 300, 271	Quercetin-*O*-hexoside-*O*-deoxyhexoside (Rutin)	*Saussurea lappa*	Flavonoid	[45]
18.	19.26	301	303	302	C_15_H_10_O_6_	240, 141	Hesperetin	*Saussurea costus*	Flavonoid	[7]
19.	25.42	-	611	610	C_27_H_30_O_16_	448, 285,267, 175	Luteolin-di-*O*-hexoside	*Saussurea costus*	Flavonoid	[7]
20.	27.69	369	371	370	C_16_H_18_O_10_	207, 192	Fraxetin-*O*-hexoside	*Saussurea eopygmaea*	Coumarin	[49]

### Flavonoids

As demonstrated in Table 1, flavonoids constituted the predominant category of identified compounds in the investigated extract. A total of thirteen flavonoids were identified by comparison and matching their fragmentation pattern with those described previously: chrysoeriol-*O*-hexouronide (**5**) [7], nepetin-*O*-hexoside (**6**) [44], isorhamnetin-*O*-hexoside-*O*-deoxyhexoside (**9**) [7, 44], diosmetin-*O*-hexoside (**11**) [1, 7], apigenin-*O*-hexoside (**12**) [45], gossypetin-*O*-hexoside (**13**) [7], hispidulin (**14**) [46], acacetin-*O*-hexoside-*O*-deoxyhexoside (**15**) [7], apigenin (**16**) [7], quercetin-*O*-hexoside-*O*-deoxyhexoside (**17**) [45], hesperetin (**18**) [7], and luteolin-di-*O*-hexoside (**19**) [7]. We noticed that the hexose and rutinose moieties of identified flavonoids were the most common. The MS/MS fragmentation analysis showed loss of a hexose moiety (*m/z* 180), followed by loss of rhamnose moiety (*m/z* 164) to produce an intense peak corresponding to aglycone with high abundance in their fragmentation pattern. Different phytochemical studies have proven isolation of identified flavonoid aglycones and their hexosides, as well rutinosides from different *Saussurea* extracts such as *S. involucrate*, *S. Lappa*, *S. tridactyla* and *S. stella* [44, 47]. The number and structure of bound sugar moieties, as well glycosidic linkages, affect the MS and MS-MS spectra of flavonoid glycosides [48]. The loss of well-defined mass fragments from the pseudomolecular ion may provide detailed data about the related saccharide. The cleavage of the sugar bond initiates fragmentation of glycoside, and this cleavage identifies the aglycone.

#### Lignans

According to the previous research reports, different lignan structures with various biological activities were previously reported in *Saussurea* extracts [1]. Similarly, the current study revealed identification of three lignans namely, medioresinol (**2**), syringaresinol (**7**), pinoresinol-*O*-hexoside (**8**) based on HPLC-MS/MS of the observed deprotonated pseudomolecular ions.

### Coumarins

Two compounds of coumarin class were identified in the investigated extract. First compound is isopimpinellin (**1**) which was previously isolated from *S. involucrate* [3]. The other compound is coumarin glycoside identified as fraxetin-*O*-hexoside (**20**), previously obtained from *S. eopygmaea* [49].

### Other compounds

Anthocyanidin compound was identified as procyanidin B2 (**4**) in the extract [7]. Another compound of sesquiterpene lactone, namely costunolide (**10**) was identified and previously isolated from the same extract [45].

### Assessment of cytotoxic activity using MTT assay

**Fig. 2:**
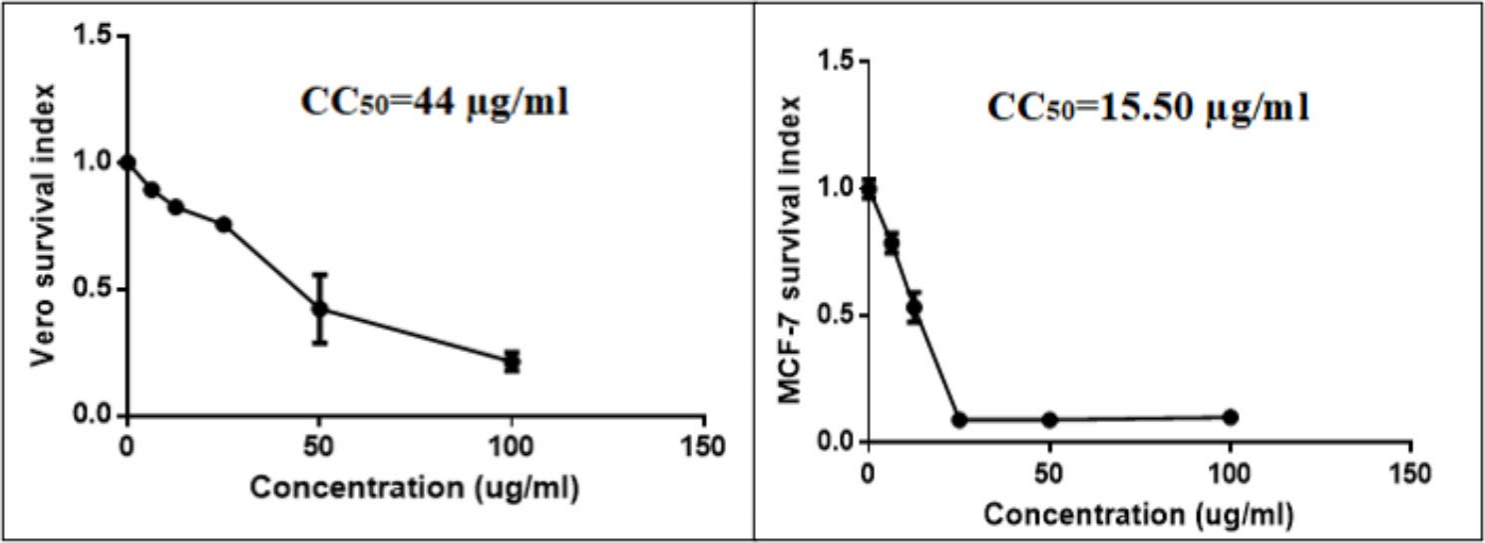
CC_50_ Values of *D. costus* extract on Vero cells (A) and MCF-7 (B) using MTT assay

The cytotoxic effect of various concentrations of plant extract (100, 50, 25, 12.5, and 6.25 µg/mL) revealed that its CC_50_ (the highest dilution of the tested material that kills 50% of the cells) was detected at concentration of 15.50 and 44 µg/mL, on MCF-7 and Vero cells, respectively, after 48 h of cell exposure (Fig. 2). According to the National Cancer Institute guidelines, the plant extract and/or a compound with CC_50_ values < 20 µg/ml is considered as cytotoxic active agent [27]. Accordingly, the tested extract is considered as anti-cancer agent. In agreement with our results, the ethanol plant extract was found to inhibit proliferation of gastric carcinoma using gastric AGS cancer cells in a dose and time-dependent manner [50]. Grippingly, some of the phytoconstituents identified in the extract (Fig. 1) have already been confirmed to possess anticancer activities for instance, isorhamnetin glycoside isolated from *Opuntia Ficus-indica* pads was reported to inhibit two different human colon cancer cells (HT-29 and Caco2) by 4.90 and 8.20 µg/mL, respectively [51]. In breast cancer tissues, Lysine-specific demethylase 1 (LSD1) has emerged as a therapeutic target for cancer as it is highly expressed in oestrogen receptor and progesterone receptor-negative tumours initiating tumour differentiation, proliferation, metastasis, and invasion [52]. Among identified compounds in our study, diosmetin glycoside and hesperetin were found to inhibit LSD1 by IC_50_ values of 21.83 and 78.76 µM, respectively [53]. Costunolide was reported to suppress tumor growth as well as metastases of human breast cancer cells via TNF-*α*-induced NF-κB activation, leading to inhibition of triple-negative breast cancer cell line (MDA-MB-231) migration and invasion. Further, it resulted in reduced expression of matrix metallopeptidase (MMP)-9 which is a well-known NF-κB-dependent gene and play a vital role to proceed breast cell cancer growth and metastases [21, 22]. In another study, costunolide showed inhibitory effect on a human monocyte cell line THP-1 using reporter gene assay which was induced by a tumor-promoting phorbol ester 12-O-tetradecanoylphorbol-13-acetate (TPA) used for tracing out the promoter activity of the inducible nitric oxide synthase (iNOS) gene [54]. iNOS activity was boosted by TPA which in turn inhibited by costunolide (IC_50_ = 2 mM). Also, costunolide exhibited inhibitory effects on the telomerase activity of MCF-7, and MDA-MB-231 cells in a concentration and time-dependent behaviour and such inhibition was demonstrated in the expression of hTRET mRNA [55]. Rutin, as another extract component, was found to possesses anti-tumor activities by triggering apoptosis in triple-negative breast cancer and upregulation of apoptosis signal-regulating kinase-1 (ASK1) and c-Jun N-terminal kinase (JNK) [56]. Luteolin-*O*-glucoside as main constituent of *Cuminum cyminum* demonstrated potent inhibitory effect against MCF-7 cell line with IC_50_ value of 3.98 µg/mL and selectivity index (SI) of 8.0 [57].

### Assessment of apoptotic effect against MCF-7 using quantitative RT-qPCR assay

To confirm that the extract inhibits the proliferation of MCF-7 cells, we investigated the apoptotic effect using RT-qPCR analysis. The results showed that mRNA expressions of Caspase 3, BCl2 and Bax genes were upregulated after 24 and 96 h of cell exposure to the plant extract (Table 2). This observation indicated that the apoptotic effect might be mediated through classical intrinsic pathway but further insightful analysis on other apoptotic genes profile and cell cycle analysis is needed to identify the exact possible targets which affect proliferation of human breast cancer cells. There are three major apoptosis-linked pathways such as mitochondrial/apoptosome pathway, the death receptor pathway, and the CTL/NK-derived granzyme B-dependent pathway; all these pathways resulted in caspase activation [58]. Caspase activation terminates during cell apoptosis and the inflammatory response [58]. Mitochondrial apoptosome-driven caspase activation represents the key to caspase-activating mechanism initiated by cytotoxic drugs and leads to caspase-2, -3, -6, -7, -8, -9, and -10 activation during apoptosis [58]. Caspases-3 and -9, are considered the most important parameters during mitochondrial apoptosome-driven caspase activation. The intrinsic mitochondrial pathway of apoptosis is strictly controlled by Bcl-2 family proteins, incorporating Bcl-2, Bcl-XL and MCL-1 [59]. There is strong association between Bcl-2 and Bax to regulate apoptosis [60].

**Table 2:** Quantitation of apoptotic genes expression after 24 and 96 h of cell exposure (Caspase, Bax and anti-apoptotic BCl2) on a transcriptional level

Treatment	Caspase 3 RQ 2^−∆∆CT^		Bcl2 RQ 2^−∆∆CT^		Bax RQ 2^−∆∆CT^	
	24 h	96 h	24	96 h	24 h	96 h
Cell control	1	1	1	1	1	1
*D. Costus*	13.1	822	1.8	824	2.1	213
Paclitaxel	17.3	325	2.4	44.9	5	164.2

IF RQ < 1 it is down regulation, > 1 up-regulation

### Assessment of antiviral activity against human adenovirus type 5

The results showed that the plant extract (25 µg/ml) exerted antiviral activity via adsorption, and virucidal mechanisms, as evidenced by undetected levels of viral copies using qRT-PCR assay. It could prevent viral entry into host cells, as well as preventing viral infectivity after incubation with the virus for 1 h at 4 °C. In the line of our results, medioresinol (as one of identified compounds of extract) isolated from *Crataegus cuneate*, was found to suppress hepatitis C virus (HCV) production in a dose-dependent manner via inhibition of HCV-RNA replication but not viral entry or translation [61]. Procyanidin B2 obtained from *Cassia fistula* potentially inhibited coronavirus disease protease [62] and human immunodeficiency virus (HIV) [63]. Apigenin selectively blocks Enterovirus-71 infection by disrupting viral RNA association with hnRNP A1 and A2 proteins with IC_50_ and CC_50_ values of 10.3 and 79.0 µM, respectively [64]. Further, apigenin was found to have anti-adenoviral activity against three viral types including Adv3, Adv-8 and Adv-11 with EC_50_ values in the range of 8.0 to 26.4 mg/L [65]. This exploratory data will encourage further in vitro study to obtain therapeutic index and then initiate in vivo study.

## Conclusions

We identified the chemical profile of *D. costus* root extract through HPLC/ESI-MS/MS analysis. Twenty phytoconstituents were tentatively identified and characterized as belonging to flavonoids being the major category along with lignans, coumarins, and anthocyanidin. Biological evaluation showed that *D. costus* is a promising candidate against proliferation of human breast cancer cells, but such observation needs further insights on the molecular level to elucidate the interplay between apoptotic genes expression and their role in cancer cell death and in vivo studies before its application on a clinical setting. In addition, its antiviral activity against one of the common respiratory infections like adenovirus increases its value in biomedical application and inspires its targeting application in a nano-based formulations.

### Electronic supplementary material

Below is the link to the electronic supplementary material.

Supplementary Material 1

## Data Availability

Data are available upon request from the first author, Heba A. S. El-Nashar; heba_pharma@pharma.asu.edu.eg.

## Abbreviations

Adv-5: Human Adenovirus Type 5
BAX: Bcl-2-associated X protein
ELISA: Enzyme-linked immunosorbent assay
HPLC-ESI-MS/MS: High-performance liquid chromatography/​electrospray ionization mass spectrometry
MCF-7: Breast cancer cells
MMP-9: Matrix metallopeptidase 9
MTT: 3-(4,5-dimethylthiazol-2-yl)-2,5-diphenyltetrazolium bromide
NF-κB: Nuclear factor-kappa
RT-qPCR: Quantitative reverse transcriptase-polymerase chain reaction
TIC: Total ion chromatogram
TNF-α: Tumor necrosis factor-alpha
