# Supplementary material for: HPLC-ESI/MS-MS characterization of compounds in Dolomiaea costus extract and evaluation of cytotoxic and antiviral properties: molecular mechanisms underlying apoptosis-inducing effect on breast cancer
Source: BMC Complement Med Ther. 2023 Oct 6;23:354. doi: 10.1186/s12906-023-04164-9 (PMC10559653; doi:10.1186/s12906-023-04164-9)
Supplement: Supplementary file 1 — Supplementary Material 1 [file 12906_2023_4164_MOESM1_ESM.docx]

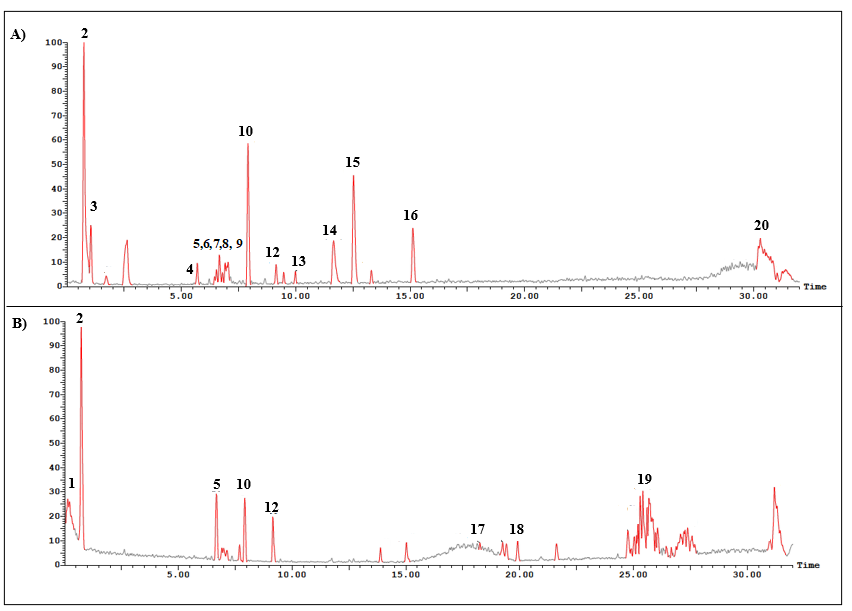
**Supporting information**

**Fig S1**. Total ion chromatogram (TIC) for 70% ethanol extract of *D. costus* in negative (A) and positive (B) ionization modes.
